# Insights into the Role of Biopolymer-Based Xerogels in Biomedical Applications

**DOI:** 10.3390/gels8060334

**Published:** 2022-05-29

**Authors:** H. P. S. Abdul Khalil, Esam Bashir Yahya, Husnul Azan Tajarudin, Venugopal Balakrishnan, Halimatuddahliana Nasution

**Affiliations:** 1 School of Industrial Technology, Universiti Sains Malaysia, Penang 11800, Malaysia; essam912013@gmail.com (E.B.Y.); azan@usm.my (H.A.T.); 2 Cluster of Green Biopolymer, Coatings and Packaging, School of Industrial Technology, Universiti Sains Malaysia, Penang 11800, Malaysia; 3 Institute for Research in Molecular Medicine, Universiti Sains Malaysia, Penang 11800, Malaysia; venugopal@usm.my; 4 Department of Chemical Engineering, Faculty of Engineering, Universitas Sumatera Utara, Medan 20155, Indonesia; halimatuddahliana@usu.ac.id

**Keywords:** xerogel, biopolymers, dried gels, porous materials, biomedical applications

## Abstract

Xerogels are advanced, functional, porous materials consisting of ambient, dried, cross-linked polymeric networks. They possess characteristics such as high porosity, great surface area, and an affordable preparation route; they can be prepared from several organic and inorganic precursors for numerous applications. Owing to their desired properties, these materials were found to be suitable for several medical and biomedical applications; the high drug-loading capacity of xerogels and their ability to maintain sustained drug release make them highly desirable for drug delivery applications. As biopolymers and chemical-free materials, they have been also utilized in tissue engineering and regenerative medicine due to their high biocompatibility, non-immunogenicity, and non-cytotoxicity. Biopolymers have the ability to interact, cross-link, and/or trap several active agents, such as antibiotic or natural antimicrobial substances, which is useful in wound dressing and healing applications, and they can also be used to trap antibodies, enzymes, and cells for biosensing and monitoring applications. This review presents, for the first time, an introduction to biopolymeric xerogels, their fabrication approach, and their properties. We present the biological properties that make these materials suitable for many biomedical applications and discuss the most recent works regarding their applications, including drug delivery, wound healing and dressing, tissue scaffolding, and biosensing.

## 1. Introduction

In the past few years, we have witnessed the development of various novel functional materials from different precursors. Xerogels and aerogels are two examples of porous, structured materials that result from the different drying techniques of wet gels [1]. The attractive and unique properties of such porous materials arise from the extraordinary flexibility and resilience of the sol–gel developing process, which is combined with either ambient drying (xerogel) [2] or supercritical drying (aerogel) [3]. These materials have been prepared from several precursors, including silica [4], carbon [5], synthetic [6], and biopolymers [7]. Biopolymeric xerogels possess different physical, chemical, mechanical, and biological properties, depending on several factors, including precursor material/s, solvent medium, and drying conditions [7]. These factors also influence the shrinking of the biopolymeric gels, leading to an increased density and reduced porosity [8].

The structure, shape, and morphology of xerogels can be controlled in both the synthesizing and drying phases, but their porosity remains less than that of aerogels of the same materials [9]. Recently, xerogels have been widely synthesized from biopolymeric materials such as cellulose, chitosan, alginate, and pectin [10,11,12,13,14,15]. Such precursor materials are known for their biocompatibility and non-toxicity, which make them suitable for many biomedical applications, such as drug delivery, wound healing and dressing, tissue scaffolding, and biosensing applications [16]. Xerogels of the same material differ from aerogels in terms of their shrinking ratio, density, porosity, and specific surface area [8,17]. Although aerogels are higher in porosity and specific surface area, the synthesis of the xerogels under ambient pressure drying, which does not require energy-consuming instruments, has made the xerogels desirable materials, especially in the cases of operational risks and economic issues [18]. The intermediate porosity of xerogels is highly preferable in sustained drug release; the high porosity of aerogels may lead to fast drug release, which is not desirable in some cases, such as cancer drug therapy [19,20]. Several research papers have been recently published regarding the fabrication and characterization of biopolymeric xerogels for different medical and biomedical applications [21,22,23], which are increasing by the day. However, to the best of our knowledge, a limited number of review papers on the applications of biopolymeric xerogels in biomedical applications have been published [24,25]. Salimian et al. [26] generally reviewed aerogel/polymer nanocomposites, and our previous reviews also regarded the applications of aerogels, but not xerogels, for biomedical applications [27]. In this review, we present, for the first time, an overview of the xerogels, their fabrication approach, and their properties, including the biological properties that make these materials suitable for many biomedical applications. We also highlight the most recent works regarding the biomedical applications of biopolymeric xerogels, including utilizing them for drug delivery, wound healing and dressing, tissue engineering applications, and the development of smart biosensors.

## 2. Xerogel Functional Material

A xerogel is defined as a porous, structural material that can be obtained via the evaporative drying of any precursor’s wet gel. Although the porosity and surface area of xerogels are lower than the aerogels, they are characterized by their easy and unexpansive fabrication, better mechanical stability, and higher density compared with aerogels [28].

### 2.1. Fabrication Techniques

**Figure 1:**
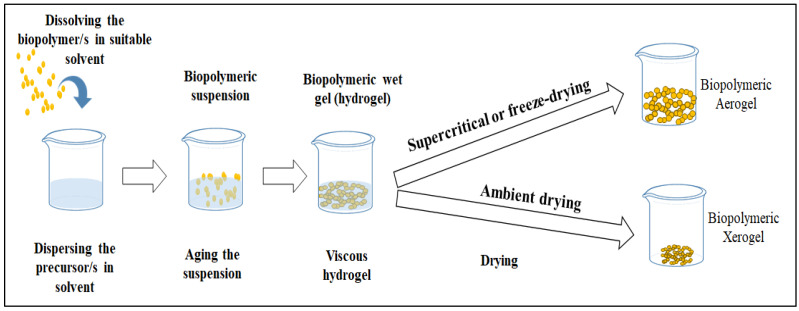
Schematic drawing of biopolymeric xerogel fabrication process and the difference between xerogels and aerogels.

The fabrication of xerogels generally consists of forming the polymeric hydrogel and drying that hydrogel in a way that retains (at least in part) its porous texture after the drying [29]. The process varies from one polymer to another, and drying conditions also differ based on the used solvent and precursor material/s. Pectin xerogel has been prepared from its alcogel. The authors used meld temperature (60 °C) for the drying purpose under vacuum conditions for 4 days until the complete drying of the alcogel [12]. The authors reported that in order to prevent a major collapse during the drying process, ionic gelation is a necessary step. A massive shrinkage of around 90 vol% commonly occurs after evaporative drying due to structural collapse, leading to an increase in the density of the material and a reduction in its porosity. The attractive properties of the biopolymeric porous hydrogels arise from their extraordinary flexibility during the sol–gel phase, which is mostly combined with various drying techniques, leading to the formation of the desired xerogel. Cellulose xerogel has been fabricated using a facile approach consisting of three steps: the partial ionic liquid dissolution of cellulose suspension, non-solvent rinsing, and drying [30]. In a different study, cellulose nanofiber xerogels were fabricated by Toivonen et al. [31] through a solvent exchange process (with octane), the vacuum filtration of their solvent dispersion, and finally, ambient drying. The authors reported a mesoporous xerogel with good porosity and surface area. Melone et al. [32] suggested a new, economically affordable synthesization protocol for the design of novel xerogels based on the cross-linking of TEMPO-oxidized cellulose nanofibers (TOUS-CNFs) and branched polyethyleneimine. The xerogel exhibited high adsorption capability for different organic pollutants, indicating its potential for water decontamination. In a different work, the authors were able to prepare different xerogels with attractive properties by cross-linking TEMPO-oxidized and ultra-sonicated cellulose nanofibers [33]. The drying step is the most important in most cases of biopolymeric xerogel fabrication. It directly affects most of the physical and morphological properties of the material. Xerogels and aerogels are the two closest relatives of polymeric substances, with slight differences in terms of fabrication approaches and properties. Unlike aerogels, xerogels cannot be formed from pure nanocellulose or any non-gel forming polymers [27,34]. Such biopolymers require cross-linking in order to form gels in them, then drying these gels to obtain xerogels [35]. Chitosan-silica xerogel was prepared by sol-gel and emulsification-crosslinking [36]. The addition of 20 wt% of SiO_2_ was found to be enough to make the xerogels exhibit a regular spherical shape with sufficient dispersity and a uniform microstructure for drug delivery applications. However, compared with the pure chitosan xerogel-based microspheres, this hybrid showed significantly improved in vitro bioactivity in addition to good drug loading capacity and sustained release. Figure 1 presents an illustration of biopolymeric xerogel fabrication and the difference between biopolymeric xerogels and biopolymeric aerogels.

### 2.2. Properties and Advantages of Xerogels

**Figure 2:**
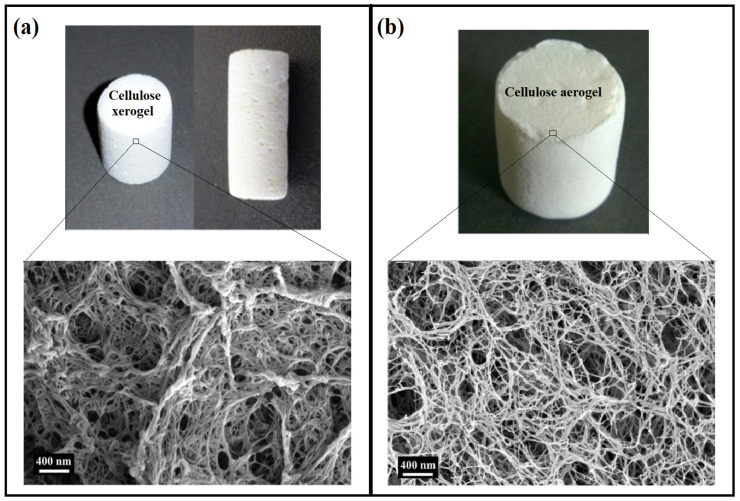
Comparison between cellulose xerogel and aerogel in term of shrinking and porosity; (**a**) present the xerogel sample of cellulose-based xerogel and the SEM image, and (**b**) present its aerogel and the SEM image. Adapted with permission from Ganesan et al. [39]. Copyright 2016 Elsevier.

A xerogel is a solid, porous material resulting from the slow drying of hydrogels at room temperature, with unconstrained shrinkage depending on the type of precursor/s. Xerogels differ from aerogels in many aspects, including their shrinkage ratio, porosity, specific surface area, and bulk density [37]. Xerogels generally possess higher shrinkage than aerogels, and thus, they have lower porosity, lower surface area, and greater bulk density. Groult et al. [12] compared the properties of pectin xerogels and aerogels and found that in order to prevent a major collapse during the drying process, ionic gelation is a necessary step. The xerogels had bulk density and porosity of 1.057 g/cm^3^ and 29.5%, respectively, compared with the pectin aerogels, which had 0.083 g/cm^3^ and 94.4% for the bulk density and porosity, respectively. The xerogels exhibited a higher loading efficiency of 94% compared with the aerogels’ loading efficiency, which was recorded to be 62%. The mechanical properties of xerogels vary depending on the type of precursor materials; in most cases, xerogels possess better mechanical properties than aerogels due to their lower porosity and higher bulk density [38]. Similarly, Ganesan et al. [39] prepared cellulose-based xerogels and aerogels and compared their characteristics, as presented in Figure 2. The authors found that the aerogels possessed significantly higher porosity, ranging between 92.7 and 96.4%, while the xerogels only possessed a porosity of 70.2 to 80.3%. The properties of biopolymeric xerogels are highly influenced by two main factors: the precursor material/s and the liquid–vapour interface, in addition to the solvent medium, which affects the drying process [13]. Thus, changing these factors will lead to xerogels with different physical and morphological properties.

Solvents such as ethanol have similar surface tension values to isopropanol, and research has reported that using these two solvents to prepare xerogel in the same condition could yield xerogels with different physical properties due to the change in vapour pressures [40]. Pramanik et al. [9] used nanocellulose in different mass ratios to improve the mechanical strength of polyvinyl alcohol xerogels. The authors reported that increasing the nanocellulose content led to a significant enhancement in the thermal properties of the xerogel. However, a xerogel rupture occurred in the case of a higher quantity of nanocellulose (18%) due to the formation of weak cellulose-rich regions. The addition of this much nanocellulose in the polymeric matrix increased the brittleness of the xerogels, which is the main cause of xerogel fracture. Silk fibroin-based xerogels possess great water absorption capacity, and Cheng et al. [23] reported that their xerogels were able to absorb up to 90 times its own mass of water within a minute in addition to its great hemostatic properties, making such material suitable for absorbing other body exudates. Several attempts have been made to produce aerogel-like xerogels under ambient conditions to minimize the shrinkage. However, the resulting xerogels in most of the cases inevitably took the form of thin films with relatively low porosity [7]. Prakash et al. [41] developed a unique approach to exchange the hydrogel’s solvent for a solvent with a lower polarity than water, such as pentane or hexane, to reduce the capillary force and thus produce xerogels with higher porosity. Other materials, such as organosilicons, have been introduced to the xerogels to enhance the optical transparency of the xerogels and make them exhibit rubbery compression [42]. Cellulose nanofiber xerogels were fabricated through a solvent exchange process with mesoporous and in a film-like shape [31]. The xerogel possessed 60% porosity and 200 m^2^/g specific surface area, which is considered close to the properties of aerogels. The characteristics of biopolymeric xerogels are highly influenced by the preparation conditions, as they directly affect the shrinkage of the hydrogels.

### 2.3. Suitability of Biopolymeric Xerogels in Biomedical Applications

Toxicity evaluation is very important when it comes to any medical applications, and the material will directly attach to the human or animal cells. Although many of the natural materials did not show significant toxicity to living cells, the preparation conditions may alter the chemistry of these materials and alter their biological effects [43,44]. Biopolymeric xerogels are dried forms of the biopolymer/s precursor; they have the chemical and biological characteristics of that biopolymer/s [45]. Several xerogels have been prepared without any need for further chemical addition or modification, but in other cases, natural compounds are added to extend the applications such as adding essential oils as an antibacterial agent. Biopolymers are known for being biocompatible and non-cytotoxic; they have been evaluated in several forms including the raw biopolymers [46], films [47], membranes [48], composites [49], hydrogels [50], aerogels [51], and even xerogels [14]. Although the number of cytotoxicity evaluations regarding biopolymeric xerogels is limited compared with aerogels, despite the drying process, aerogels and xerogels are prepared with the same principle, and thus, both of them are highly biocompatible, non-cytotoxic, and allow the attachment and migration of cells [52]. Refer to Table 1 for a summary of the cytotoxicity and biocompatibility evaluations of biopolymeric xerogels.

## 3. Biopolymeric Xerogels in Biomedical Applications

Biopolymeric xerogels are porous networks of many unique and desirable properties that have been widely studied for different biomedical applications including controlled and sustained drug delivery, wound dressing and healing applications, tissue engineering scaffolds, and other applications [23]. Owing to the biocompatibility, non-cytotoxicity, and non-immunogenicity of the biopolymers, biopolymeric xerogels are considered to be a safer option than inorganic and synthetic materials in medical applications [1,59].

### 3.1. Drug Delivery

**Figure 3:**
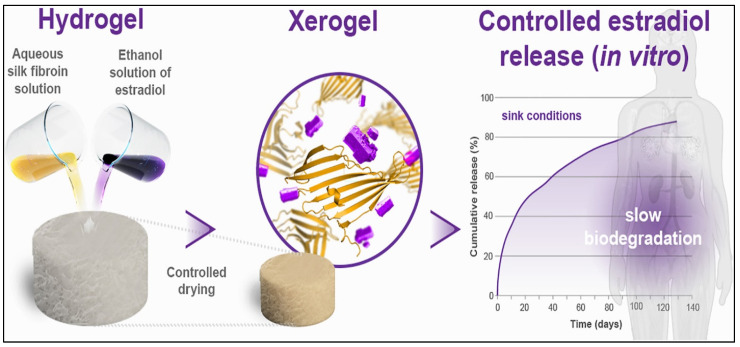
Illustration of silk fibroin based xerogels in controlled release of estradiol drug. Adapted with permission from Križman et al. [63]. Copyright 2022 Elsevier.

Xerogels have been extensively studied for their potential use in drug delivery since their discovery. Owing to their porous texture, their ability to control pore structure, and their large surface area, they attracted the attention of scientists in many pharmaceutical applications. Such desirable characteristics are favoured by drug loading and allow for better control of the drug release behavior [60]. Zhou et al. [16] used a poly (ε-caprolactone)-chitosan-silica xerogel for tetracycline hydrochloride delivery by green fabrication route. The presence of silica in the xerogel significantly enhanced the thermal stability and endowed good in vitro bioactivity and drug release behavior for the xerogel. The ability to modify the surfaces of biopolymers within the xerogel facilitates the drug incorporation in higher loading capacity and more sustained release. In a recent study, an alginate-based xerogel was modified using g-poly (methacrylic acid; AGM2S) for insulin delivery toward wound care [61]. The authors reported significant improvement in the physical stability, good swelling, and low degradation of the modified xerogel. More than 70% of loaded insulin was released from the xerogel in two days, which modulated the healing response [61]. In a different study, a novel xerogel was prepared from silica and poly(ethylene glycol) by the facile sol–gel route and showed sustained release of an enrofloxacin antibiotic drug [62]. The unique properties and facile fabrication of xerogels permit the slow release of drugs, making them a better option for sustained drug delivery applications. Different precursors consisting of naturally available diatomaceous earth microparticles have been used for the first time in xerogel fabrication [20]. Such unique xerogels were modified to enhance their drug loading capacity by using a facile sol–gel method resulting in a pH-sensitive micro drug carrier, which was evaluated for diclofenac sodium drug delivery. The authors reported a significant increase in drug loading capacity and sustained drug release fitting the zero-order model. Križman et al. [63] fabricated silk fibroin-based xerogels and evaluated their potential for long-acting hormone estradiol delivery (Figure 3). Ethanol was used in the preparation process and acted as a dissolving agent for the drug in addition to an accelerator for the gelation process. The authors were able to achieve a sustained drug release of up to 129 days from the xerogel delivery system, suggesting the great potential of such biopolymeric xerogel in the prolonged release of hydrophobic drugs.

### 3.2. Antibacterial and Wound Healing Applications

**Figure 4:**
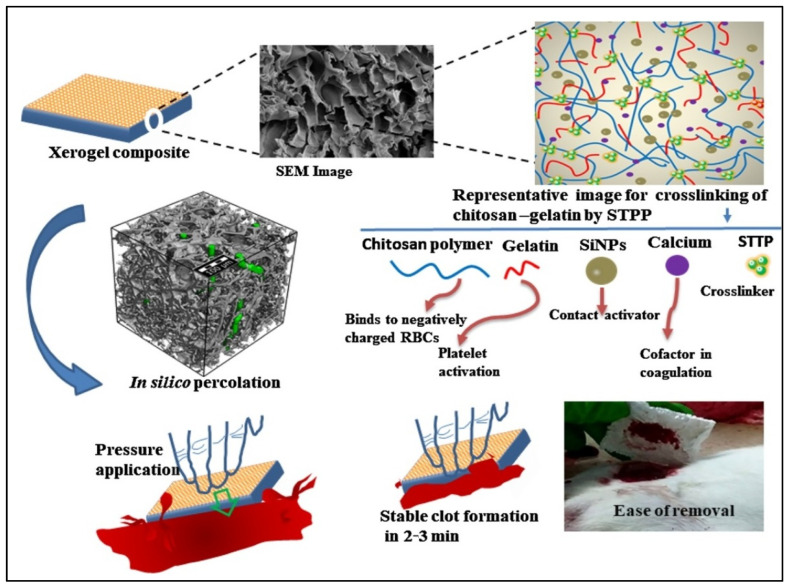
Chitosan-gelatin xerogel composite loaded with silica nanoparticles and calcium for rapid halting blood loss, showing the interaction between the biopolymers and its wound healing properties. Adapted with permission from Patil et al. [14]. Copyright 2022 Elsevier.

The process of wound healing is a complex and dynamic process consisting of several stages that lasts days or even weeks depending on multiple factors, such as the type of wound, its depth, microbial colonization, and the patient’s immune system, to enable the injured skin to restore itself [64]. Hydrogels’ antibacterial materials [65,66,67] have been widely used in wound healing applications, but they have the drawback of requiring gauze or other adjuvants to be applied to a bleeding wound. Furthermore, the overly moist environment caused by hydrogel is not conducive to promoting wound healing and the scabbing effect, especially at the early stages of wound formation [68]. Deep wounds may favor the growth of anaerobic bacteria, leading to severe inflammation and suppuration [69]. Xerogels have been used to overcome these drawbacks, which can be customized to be super-hydrophobic and/or super-adhesive functional materials [70]. In a recent investigation, Huang et al. [71] fabricated a novel xerogel with good mechanical properties, using silver nanoparticles as an antibacterial agent. The hybrid xerogel was able to rapidly capture bacteria and kill 99.9% of *E. coli* and 99.85% of *S. aureus* through the electrostatic interactions of the disulfide groups. Although silver nanoparticles have been linked with minor adverse health effects, the authors reported the good biocompatibility and non-toxicity results of the xerogel [72]. Natural antibacterial agents, such as plant essential oils and extracts, could be also loaded into the xerogel and used for wound healing. Plant polysaccharide-based xerogels are characterized by their high biocompatibility, large biodegradability, and high water absorption capacity [34]. Owing to the excessive distribution of surface functional groups, they have the potential to cross-link with natural antibacterial agents. Chitin and chitosan are the most used animal-based biopolymers in terms of wound healing application due to their special properties, including bactericidal and antifungal characteristics, high permeability to oxygen, and healing activities by stimulating fibroblast proliferation [51]. Deon et al. [73] used a silica/titania magnetic xerogel to immobilize chitosan-stabilized gold nanoparticles as an antibacterial system. Owing to the synergistic effect of chitosan and gold nanoparticles, the surface reactivity of titania, and the porous and magnetic response of silica, the xerogel system possessed strong antibacterial activity, even at an extremely low gold content. Using two or more biopolymers in xerogel fabrication was found to enhance the properties of the material and limit the shrinkage after drying; a porous xerogel was fabricated using chitosan in combination with sodium polyacrylate, polyethylene glycol wound treatment, and hemorrhage control [74]. Chitosan was used as antimicrobial agent that always cross-linked with different organic or inorganic materials, such as gelatin and tannic acid, which played a hemostatic role [75]. Gelatin is a biopolymer that is extensively used in wound and skin care applications due to its ability to activate platelet aggregation, and it can also act as an absorbable hemostatic agent [75]. Patil et al. [14] used the two biopolymers to prepare a highly porous xerogel for an efficient, multimodal topical hemostat (Figure 4). The authors ionically cross-linked gelatin and chitosan with sodium tripolyphosphate, and they were able to achieve in vitro >16-fold improved blood clotting compared to the available commercial materials. The xerogel content displayed good platelet activation and promoted the generation of thrombin, which is very important in wound healing applications. The same authors conducted an in vivo study of their xerogel on a lethal femoral artery injury and reported 2.5 min hemostasis, which is significantly faster than the commercial Gauze (4.6 min) and Celox (3.3 min), in addition to easy removal from the wound. Although xerogels have not been used for commercialization purposes yet, in the coming years, we will witness the utilization of these materials in wound healing applications, as they have great potential as topical hemostatic agents and can be used to save precious lives.

### 3.3. Tissue Engineering

**Figure 5:**
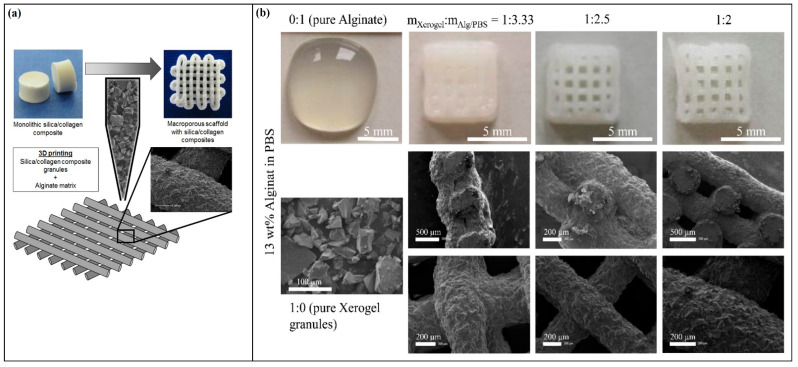
Illustration of 3D plotting approach of silica–collagen hybrid xerogel granules in an alginate matrix; (**a**) the fabrication approach, and (**b**) photographs and SEM images of a different ratio of xerogels. Adapted from Rößler et al. [79].

Porous biopolymeric xerogels have been also used in tissue engineering scaffolds, as the easy adjustment of pore size and structure, in addition to their high biocompatibility, makes them a highly favorable form of the materials in such an application. A porous chitosan/berberine hydrochloride composite xerogel was prepared for tissue regeneration and hemostatic applications [76]. This biopolymeric xerogel exhibited good antibacterial activity, hemostatic properties, and fast degradability after immersion in phosphate-buffered saline. The authors reported good biocompatibility and strong hemostatic potential, as it was only composed of natural materials, which implies that it is a promising material for skin regeneration and hemostatic applications. The unique properties of some biopolymers, such as the antimicrobial activity of chitosan and promoting cell growth in collagen and silk fibrin, made their xerogels highly favorable in tissue engineering and regenerative medicine [77]. Wu et al. [78] fabricated a novel bioactive hybrid xerogel based on silk fibroin as precursor material, silica to enhance the mechanical properties, and CaO–P_2_O_5_ to enhance the xerogel’s properties for bone regeneration applications. The authors reported excellent porosity and pore structures for their xerogel and adding the silica significantly enhanced the mechanical properties. The xerogel exhibited profound bioactivity once immersed in a simulated fluid due to the hydroxyapatite layers on its surfaces. The xerogel was biocompatible, although it showed little toxicity to MC3T3-E1 cells, which was due to the effect of silica on the cells. In a similar study, Lee et al. [58] fabricated a hybrid xerogel from calcium, silica, and collagen for bone regeneration applications. The authors used calcium to promote the bone cells’ proliferation and silica to enhance the mechanical properties of collagen. Owing to the homogenous mixing and the incorporation of silica in the collagen matrix, the xerogel did not form any by-products, and it showed excellent bioactive characteristics. The hybrid xerogel expressed a better osteoblastic phenotype than the xerogels of pure collagen and pure silica. Elshishiny & Mamdouh [56] reported the fabrication of novel tri-layered, asymmetric, porous xerogel scaffolds for skin regeneration applications. The xerogel scaffold consisted of two layers: an upper layer of electrospun chitosan–poly(vinyl alcohol) and a lower layer of their regular xerogel. The authors fixed the two layers together by using a third material, fibrin glue, as a middle layer. This novel fabrication showed promising scaffold-swelling capability in addition to a high absorption capacity in regard to wound exudates. The porosity of the xerogel provided an optimum environment for the fibroblast cells’ migration and proliferation. In a recent study, Rößler et al. [79] used the three-dimensional (3D) plotting of a silica and collagen hybrid xerogel scaffold in another biopolymeric matrix consisting of alginate (Figure 5). The authors used viscoelastic alginate as a matrix to enhance the biocompatibility and binding properties of the xerogel scaffold, and they reported that alginate concentration is the golden key to controlling the shape regularity of the xerogel granules.

### 3.4. Biosensing

**Figure 6:**
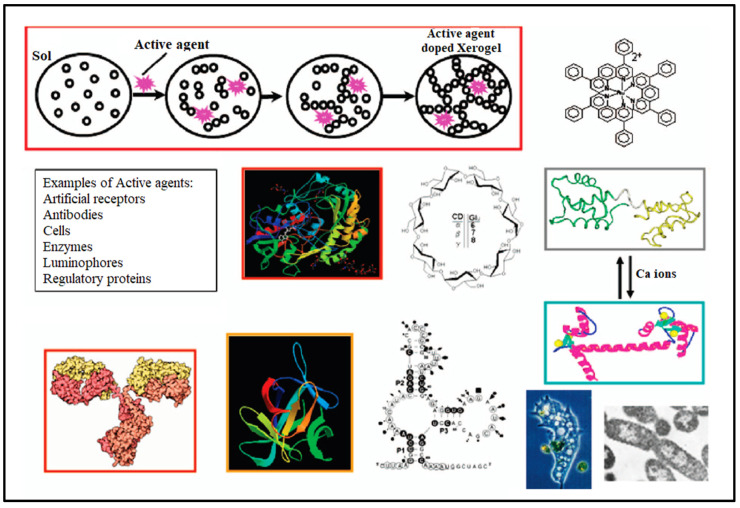
The role of active agents in the fabrication of xerogels based biosensors, presenting different examples of active agents. Reprinted with permission from Holthoff et al. [82]. Copyright 2007 American Chemical Society.

Biopolymeric xerogels have also been utilized in the sensing applications of many medically important parameters such as glucose level, uric acid, cholesterol, etc. Xerogels possess desired biosensor properties, such as porous structure and high surface area, making them highly advanced detection tools [80]. Khattab et al. [13] developed an easy-to-use, smart, microporous cellulose xerogel-based colorimetric sensor by immobilizing bromocresol purple chromophore into a cross-linked carboxymethyl cellulose xerogel matrix. Proton shifting from the hydroxyl group in the bromocresol purple dye to ammonia nitrogen enabled the identification of ammonia gas. Unlike dense material and metal-based xerogels, biopolymeric xerogels are distinguished by their non-toxicity, lighter weight, and larger surface area; thus, they are suitable for the identification of different parameters both in liquids and gaseous analytes [81]. The home-based detection and quantification of common analytes such as glucose monitoring, in addition to environmental routine monitoring, is exceedingly challenging and requires high measurement accuracy. Xerogel-based biosensors attracted attention for this purpose, as they are inexpensive, robust, and reusable materials able to meet all the requirements of biosensors [27]. The fabrication of xerogel-based biosensors began with the immobilization of active agents that are able to detect the desired parameters. Numerous active compounds, such as antibodies, active receptors, enzymes, cells, regulatory proteins, etc. have been used for this reason [82]. Figure 6 presents an illustration and several examples of active agents and their role in xerogel-based biosensors.

Three main approaches have been reported for the immobilization of active agents in the xerogels, including entrapment, physisorption, and covalent attachment [83]. The physisorption approach is the simplest, but it has the drawback of the random orientation of active agents on the xerogel, which could lead to them being unable to access the target molecule, thus lowering the accuracy of the xerogels [84,85]. To solve this issue, covalent attachment, which generally forms more stable interfaces, was developed. However, this approach also severs from the partial orientation of some kinds of active agents in addition to being a more expensive and time-consuming approach [82]. Freeman et al. [86] prepared the first generation of novel amperometric glucose biosensors, but they used several synthetic materials instead of biopolymers, which has the drawback of toxicity. To solve this issue, Alharthi et al. [87] recently used a nanocellulose acetate-based xerogel for the colorimetric detection of urea. The authors reported that their sponge-like, microporous xerogel was highly sensitive to urea because it used a urease enzyme as a catalytic agent and triarylmethane as a spectroscopic chromophore. The porous xerogel allowed for the in-situ integration of the triarylmethane probe, which enhanced the detection process and increased the accuracy of detection. Similarly, Abdelrahman et al. [88] developed a highly sensitive, reversible, and cost-effective biopolymeric xerogel for ammonia vapor detection. The microporous cellulose xerogel exhibited naked-eye colorimetric responsiveness immediately upon exposure to ammonia vapour. The application of biopolymeric xerogel in biosensing has not yet been extensively studied; limited works have been established, but we believe that these materials have great potential in regards to this application.

## 4. Challenges and Future Prospective

Biopolymeric porous materials such as xerogels and aerogels are still in their initial experimental stage in many biomedical applications. A limited number of materials entered the clinical trials, and most of them are still in the developmental and laboratory experimental phases. Although there are a significant number of studies that have proven that biopolymeric xerogels are highly suitable for biomedical applications, clinical and long-term evaluations of these materials are highly necessary before they can be commercialized. Smart and controlled delivery has been achieved in some experiments, especially in the case of cancer [89], but long-term evaluation and different cell evaluation have yet to be explored. Numerous challenges remain for other pathologies, as most of the current research focuses only on delivering specific drugs, particularly anticancer, anti-diabetic, and antimicrobial agents, and many of them involve in vitro studies or only short-term in vivo studies without considering the effect of these materials on other human bodies’ biological parameters. Toxicity and biocompatibility experiments are, in most cases, carried out by using one type of cell in simulation conditions [90], and the real conditions inside our bodies might be different, so such materials may not be as biocompatible as they seem. The full effects of these biopolymeric materials on the human body have not yet been determined. The future generation of therapeutic biopolymeric materials with antibiotics, antibodies, hormones, peptides, genes, etc. should minimize undesirable side effects, not increase them. The future of biopolymeric xerogels requires serious collaboration among worldwide researchers, different industries, and regulatory agencies to maintain and ensure the safety and effectiveness of these therapeutic platforms to evaluate the potential and the possibilities of xerogel production in adequate quantities and of adequate quality to meet the expected demands of society.

## Figures and Tables

**Table 1:** Illustration of biocompatibility and cytotoxicity studies of biopolymer-based xerogels.

Type of Xerogel	Experiment	Type of Cells	Conclusion	Ref
Chitosan-gelatin xerogel	Hemocompatibility, cytotoxicity assays	Mouse embryonic fibroblast cells	Good platelet activation, good biocompatibility, and thrombin generation activities.	[14]
Collagen-silica xerogel	Cell culture experiments	Human monocytes	The xerogel promoted the differentiation of monocytes into osteoclast-like cells.	[53]
Carbon xerogel	Cytotoxicity test	Fibroblast cell	The xerogel was biocompatible; the presence of carbon fibers increases the cell’s proliferation.	[54]
Chitosan coated mesoporous silica xerogels	Cytotoxicity assays	Mouse myoblast cells line	No obvious cytotoxicity was reported for the xerogel even after 7 days of the exposure.	[55]
Silk Fibroin Protein Xerogel	Hemostasis experiments	In-vitro and in-vivo rabbit ear	Good hemostatic properties were observed both in vitro and in vivo for the xerogel.	[23]
Chitosan–poly(vinyl alcohol) xerogel	Cytotoxicity and migration rate	Mouse embryonic fibroblast	The xerogel exhibited significant cell proliferation & migration rates and high biocompatibility.	[56]
Alginate-hydroxyapatite aerogel	Cytotoxicity, viability, and migration	Mesenchymal stem cells	Highly biocompatible, allowed attachment and migration.	[57]
Collagen–silica xerogel	Cell proliferation assay	Preosteoblast cells	Good biocompatibility and high level of osteoblast differentiation	[58]

## Data Availability

Not applicable.
